# MiR-608, pre-miR-124-1 and pre-miR26a-1 polymorphisms modify susceptibility and recurrence-free survival in surgically resected CRC individuals

**DOI:** 10.18632/oncotarget.12422

**Published:** 2016-10-04

**Authors:** Hou-Qun Ying, Hong-Xin Peng, Bang-Shun He, Yu-Qin Pan, Feng Wang, Hui-Ling Sun, Xian Liu, Jie Chen, Kang Lin, Shu-Kui Wang

**Affiliations:** ^1^ Department of Clinical Laboratory, The Second Affiliated Hospital of Nanchang University, Nanchang 330006, Jiangxi, China; ^2^ Medical School of Southeast University, Nanjing 210009, Jiangsu, China; ^3^ Department of Clinical Laboratory, Affiliated Hospital of Nantong University, Nantong 226001, Jiangsu, China; ^4^ Central Laboratory, Nanjing First Hospital, Nanjing Medical University, Nanjing 210006, Jiangsu, China

**Keywords:** miRNA, colorectal cancer, polymorphism, clinical outcome

## Abstract

Genetic variation within microRNA (miRNA) may result in its abnormal folding or aberrant expression, contributing to colorectal turmorigenesis and metastasis. However, the association of six polymorphisms (miR-608 rs4919510, miR-499a rs3746444, miR-146a rs2910164, pre-miR-143 rs41291957, pre-miR-124-1 rs531564 and pre-miR-26a-1 rs7372209) with colorectal cancer (CRC) risk, therapeutic response and survival remains unclear. A retrospective study was carried out to investigate the association in 1358 0-III stage resected CRC patients and 1079 healthy controls using Sequenom's MassARRAY platform. The results showed that rs4919510 was significantly associated with a decreased susceptibility to CRC in co-dominant, allele and recessive genetic models, and the protective role of rs4919510 allele G and genotype GG was more pronounced among stage 0-II cases; significant association between rs531564 and poor RFS was observed in cases undergoing adjuvant chemo-radiotherapy in co-dominant, allele and dominant models; moreover, there was a positive association between rs7372209 and recurrence-free survival in stage II cases in co-dominant and over-dominant models; additionally, a cumulative effect of rs531564 and rs7372209 at-risk genotypes with hazard ratio at 1.30 and 1.95 for one and two at-risk genotypes was examined in stage II cases, respectively. Our findings indicated that rs4919510 allele G and genotype GG were protective factors for 0-II stage CRC, rs7372209 and rs531564 could decrease RFS in II stage individuals and resected CRC patients receiving adjuvant chemo-radiology.

## INTRODUCTION

Accumulating experimental evidence shows that microRNAs (miRNAs) are crucial expression micromanagers of genes associated with colorectal cancer (CRC) [1–3]. They can post-transcriptionally destabilize the target gene and inhibit translation of the protein by specifically binding to the target mRNAs 3′-untranslated region (UTR) [4]. A large number of aberrant miRNAs were detected and validated in CRC cell lines and biopsies, suggesting that they might play roles as oncogene or tumor suppressor in carcinogenesis and progression of CRC [5–6]. However, genetic and epigenetic causes can interfere with miRNAs biogenesis or change their secondary structure, leading to abnormal expression of miRNAs or its target genes involved in CRC [7].

Recently, several studies reported association of polymorphisms within miRNA and susceptibility, drug response and survival of CRC [8–10]. However, these studies were performed in heterogeneous population with small sample size, leading to contradictory conclusions with low statistic power. Genotype CC of rs2910164 within pre-miR-146a was reported to associate with a decreased risk of CRC in 353 cases and 540 controls in Chinese population [8]. A higher CRC risk and worse relapse-free and disease specific survival were detected in individuals harbored rs2910164 CC genotype in Korea population [9]. However, lack of the association was observed between rs2910164 and risk of CRC in a total of 621 European subjects [10].

In order to investigate the association between the six miRNA polymorphisms (miR-608 rs4919510, miR-499a rs3746444, miR-146a rs2910164, pre-miR-143 rs41291957, pre-miR-124-1 rs531564, pre-miR-26a-1 rs7372209) and CRC risk, therapeutic response and clinical survival, we conducted this study in 1358 TNM 0-III stage CRC patients and 1079 healthy check-up individuals and followed-up the cases from diagnosis to the 3-years' deadline time to understand the involvement of them in CRC initiation and progression in Chinese population.

## RESULTS

### Clinical characteristics

The baseline characteristics of cases and controls were descripted in Supplementary Table S1. All of the enrolled patients were clinical confirmed TNM 0-III stage CRC cases (0 stage: 19; I stage: 172; II stage: 592; III stage: 575). Rectum distribution of the cases was 57.29%, the others were colon cancer (proximal colon: 21.72%; distal colon: 20.99%), and only 12.59% of the patients were confirmed as poor cell differentiation. All of the cases underwent curative surgery, 86.82% and 22.83% of them received adjuvant 5-fluorouracil (5-FU) based chemotherapy and radiotherapy, respectively. However, no significant difference was observed between cases and controls in age, gender, smoking, drinking, diabetes and hypertension.

### Susceptibility to CRC

Genotypes of the selected single nucleotide polymorphisms (SNPs) were detected using polymerase chain reaction(PCR)-based MassASSAY deletion system in 1358 cases and 1079 controls. The results between the polymorphisms and susceptibility to CRC were listed in Supplementary Table S2. *P*-values of Hardy- Weinberg equilibrium (HWE) of the polymorphisms excluding rs3746444 in two groups were higher than 0.05, indicating that only genotype distribution of rs3746444 within miR-499a was inconsistent with HWE. The results of random selected samples detected by PCR-bassed MassArray and DNA sequencing were in complete agreement. Rs4919510 within miR-608 was significantly associated with a decreased susceptibility to CRC in co-dominant (*p<*0.01, adjusted OR=0.70, 95%CI=0.55-0.88 for GG vs. CC), allele (*p<*0.01, adjusted OR=0.85, 95%CI=0.75-0.95) and recessive (*p<*0.01, adjusted OR=0.68, 95%CI=0.56-0.86) models. Stratified analysis showed that significant associations were only observed in stage 0-II subgroup in co-dominant (*p<*0.01 for GG vs. CC), allele (*p<*0.01), recessive (*p<*0.01) and over-dominant (*p<*0.01) models (Table 1). Whereas, no significant association was observed between the other polymorphisms and susceptibility to CRC.

**Table 1:** The association of miR-608 rs4919510 and susceptibility to CRC in overall and stratified populations

Locus	Genetic model	Genotype	Cases			Controls	*P*-value*		
			Total	0-II stage	III stage		[1]	[2]	[3]
Rs4919510	Co-dominant	CC	423	248	175	313	1.00	1.00	1.00
		CG	690	427	263	512	0.98	0.63	0.48
		GG	232	103	129	250	**<0.01**	**<0.01**	0.56
	Allele	C	1536	923	613	1138	1.00	1.00	1.00
		G	1154	633	521	1012	**<0.01**	**<0.01**	0.54
	Dominant	CC	423	248	175	313	1.00	1.00	1.00
		CG/GG	922	530	392	762	0.22	0.20	0.46
	Recessive	CC/CG	1113	675	438	825	1.00	1.00	1.00
		GG	232	103	129	250	**<0.01**	**<0.01**	0.82
	Over-dominant	CC/GG	655	351	304	563	1.00	1.00	1.00
		CG	690	427	263	512	0.07	**<0.01**	0.63

*P*-value*: [1]: *p*-value of overall cases vs. controls; [2]: *p*-value of 0-II stage cases vs. controls; [3]: *p*-value of III stage cases vs. Controls; the bold highlighted results showed statistical significance.

### Clinical response to 5-FU based chemotherapy

In this study, we investigated the associations between the selected SNPs and clinical therapeutic response to 5-FU based chemotherapy in 276 cases who received neoadjuvant chemotherapy. As a result, 2, 46, 161 and 67 were estimated as complete response (CR), partial response (PR), stable disease (SD) and progressive disease (PD) in these patients, respectively (Supplementary Table S1). Significant association was observed between pre-miR-124-1 rs531564 and objective response rate (ORR) in CRC patients (*p*=0.04, crude OR=0.42, 95%CI=0.18-0.99 for CG vs. GG; *p*=0.04, crude OR=0.41, 95%CI=0.17-0.96 for CG vs. CC/GG), however, no significant association was examined between them (adjusted OR=0.54, 95%CI=0.22-1.31 for CG vs. GG; adjusted OR=0.52, 95%CI=0.22-1.27 for CG vs.CC/GG) when it was adjusted by age, gender, smoking, drinking, hypertension and diabetes, no association was observed between the other loci and clinical response to 5-FU based chemotherapy in CRC patients (Supplementary Table S3).

### Survival of the cases

**Figure 1:**
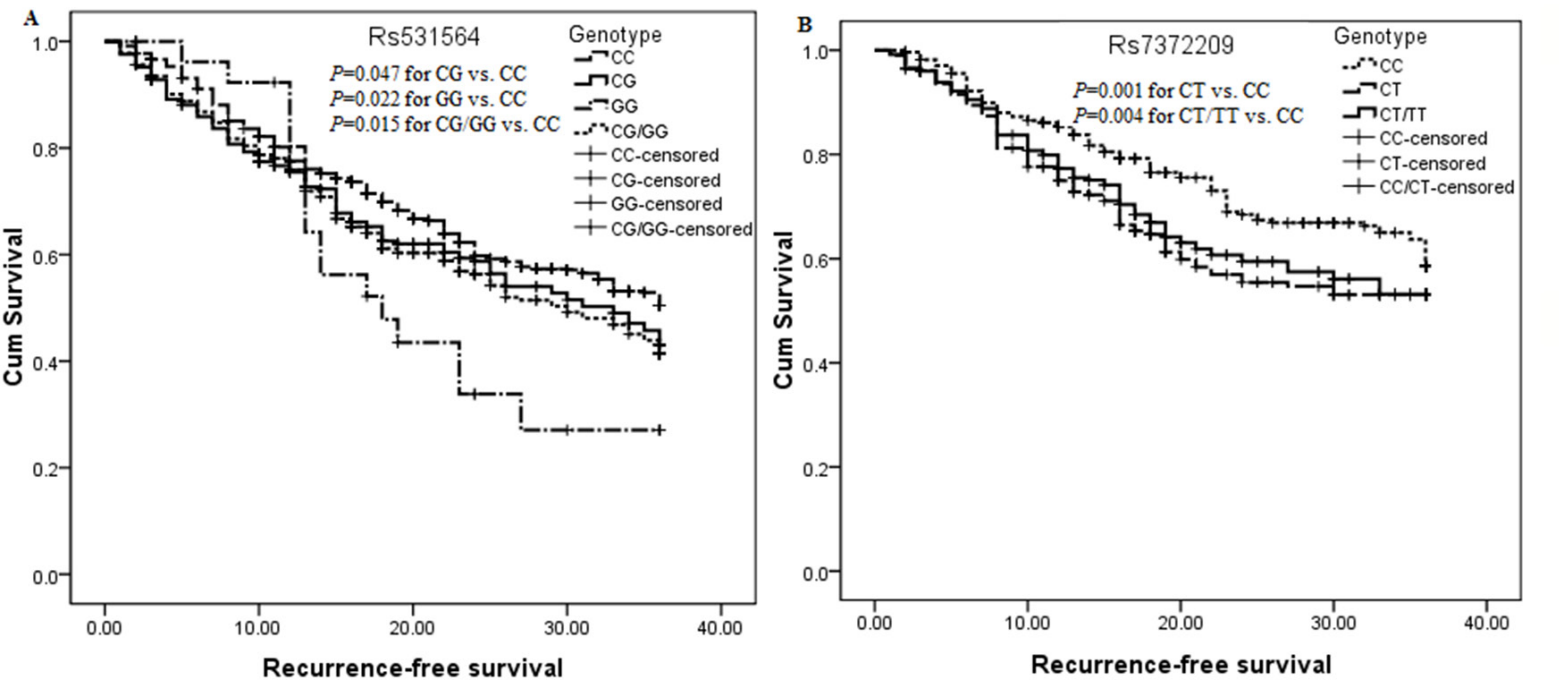
Kaplan-Meier curve analysis of the significant association between pre-miR-124-1 rs531564, pre-miR-26a-1 rs7372209 and RFS in surgically resected CRC patients **A.** Survival analysis in surgically resected CRC patients undergoing 5-FU based chemo-radiotherapy; **B.** Survival analysis in stage II surgically resected CRC patients.

Twelve hundreds and eighteen surgically resected CRC patients were selected to investigate the effect of six polymorphisms on recurrence-free survival (RFS) and 3 years' overall survival (OS), and the lost of follow-up rate in our study was 19.22%. A total of 524 cases was observed to local and distal recurrence, and 336 patients were dead in the follow-up interval (Supplementary Table S1). The median survivals of RFS and OS were 19 and 26 months, respectively. We observed rs531564 within pre-miR-124-1 (adjusted HR=1.25, 95%CI=1.02-1.52 for CG vs. CC; adjusted HR=1.26, 95%CI=1.07-1.48 for G vs. C; adjusted HR=1.28, 95%CI=1.05-1.54 for CG/GG vs.CC; adjusted HR=1.22, 95%CI=1.00-1.49 for CG vs. CC/GG) and rs7372209 within pre-miR-26a-1 (adjusted HR=1.20, 95%CI=1.00-1.42 for CT vs. CC/TT) were significantly associated with poor RFS, rather than OS (Figure 1 and Supplementary Table S4). Results of stratified analysis showed that rs531564 was associated with a short RFS in stage 0-I, II and III subgroups and in patients receiving adjuvant chemo-radiotherapy (Table 2 and Supplementary Table S5). Whereas, a worse RFS was observed only in stage II CRC patients harbored rs7372209 genotype CT and CT/TT in comparison with genotype CC (Table 2). Whereas, we didn't examined significant association between rs531564 or rs7372209 and clinical pathological characteristics (Supplementary Table S6), no association of the other polymorphisms with clinical survival was observed in these patients (Supplementary Table S4).

**Table 2:** Pre-miR-124-1 rs531564, pre-miR-26a-1 rs7372209 and RFS in subgroups stratified by TNM stage

Locus	Genetic model	Comparison	Recurrence-free survival					
			0-I stage		II stage		III stage	
			K-M	HR and 95%CI	K-M	HR and 95%CI	K-M	HR and 95%CI
Rs531564	Co-dominant	CG vs.CC	**<0.01**	**2.65(1.52-4.62)**	0.97	1.05(0.78-1.41)	0.11	1.24(0.98-1.56)
		GG vs. CC	-	-	**<0.01**	**2.01(1.15-3.53)**	0.16	1.49(0.79-2.80)
	Allele	G vs. C	**0.08**	**1.68(1.07-2.65)**	**0.05**	1.23(0.98-1.55)	**0.04**	**1.24(1.02-1.51)**
	Dominant	GC/GG vs.CC	**<0.01**	**2.37(1.36-4.13)**	0.33	1.15(0.88-1.51)	0.06	**1.26(1.01-1.57)**
	Recessive	GG vs. CC/CG	-	-	**<0.01**	**2.05(1.18-3.57)**	0.21	1.43(0.76-2.68)
	Over-dominant	CG vs. CC/GG	**<0.01**	**2.78(1.60-4.84)**	0.81	0.99(0.74-1.33)	0.12	1.22(0.97-1.54)
Rs7372209	Co-dominant	CT vs. CC	0.25	1.09(0.60-1.99)	**<0.01**	**1.56(1.21-2.00)**	0.66	0.92(0.75-1.13)
		TT vs. CC	-	-	0.95	0.95(0.59-1.54)	0.20	0.69(0.42-1.13)
	Allele	T vs. C	0.71	0.89(0.54-1.46)	0.06	1.17(0.97-1.41)	0.29	0.89(0.75-1.04)
	Dominant	CT/TT vs. CC	0.39	0.99(0.54-1.82)	**<0.01**	**1.42(1.12-1.80)**	0.45	0.89(0.73-1.09)
	Recessive	TT vs. CC/CT	-	-	0.42	0.75(0.48-1.17)	0.23	0.71(0.44-1.16)
	Over-dominant	CT vs. CC/TT	0.19	1.18(0.65-2.15)	**<0.01**	**1.60(1.25-2.04)**	0.84	0.95(0.78-1.16)

**Abbreviations:** K-M: Kaplan-Meier curve; HR and 95%CI (hazard ratio and 95% confidential interval): adjusted by gender, age, smoking, drinking, and hypertension as well as diabetes; the bold highlighted results showed statistical significance.

### Cumulative effects of unfavorable genotypes on RFS

**Figure 2:**
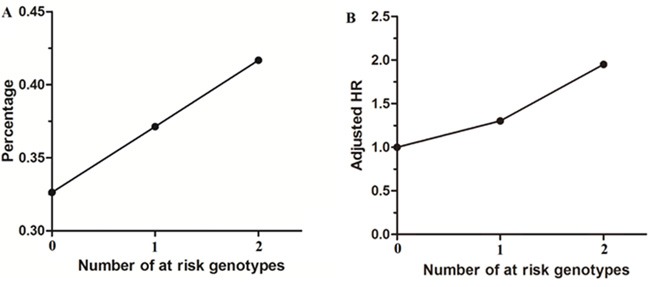
Recurrence frequency and adjusted HR in stage II surgically resected CRC subgroup according to number of at-risk genotypes **A.** Recurrence frequency of at-risk genotypes in stage II surgically resected CRC subgroup; **B.** Adjusted HR according to the number of at-risk genotype.

An unfavorable genotype analysis within rs531564 and rs7372209 was performed to examine the cumulative effect of the significant polymorphisms on RFS in stage II CRC patients. 62(32.63%), 101(37.13%) and 25(41.67%) of patients were recurrent in stage II patients who harbored 0, 1 and 2 at-risk genotypes and the median RFS of each group was 23.5, 19.0 and 16.5 months, respectively. Comparing with patients carrying the zero at-risk genotype (genotype CC of rs531564 and genotype CC of rs7372209), those who harbored one and two at-risk genotypes had about 1.30 (*p*=0.04, adjusted HR=1.30, 95%CI=1.01-1.70) and 1.95 (*p<*0.01, adjusted HR=1.95, 95%CI=1.27-2.98) folds increased risk for CRC recurrence, respectively (Table 3 and Figure 2).

**Table 3:** Number of at-risk genotypes within rs531564 and rs7372209 and RFS in 522 stage II surgically resected CRC patients

Number of at-risk genotypes	Patients		Median survival	*P*-value of K-M	HR and 95%CI	
	Overall	Recurrence	Months		[1]	[2]
0	190(36.40%)	62(32.63%)	23.50	1.00	1.00	1.00
1	272(52.11%)	101(37.13%)	19.00	**0.04**	**1.36(1.02-1.73)**	**1.30(1.01-1.70)**
2	60(11.49%)	25(41.67%)	16.50	**<0.01**	**1.72(1.13-2.62)**	**1.95(1.27-2.98)**

**Abbreviations:** K-M: Kaplan-Meier curve; HR: hazard ratio; 95%CI: 95% confidential interval; [1]: crude HR and 95%CI; [2]: adjusted by gender, age, smoking, drinking, hypertension and diabetes; the bold highlighted results showed statistical significance.

### *In silico* prediction of SNP on miRNA folding

**Figure 3:**
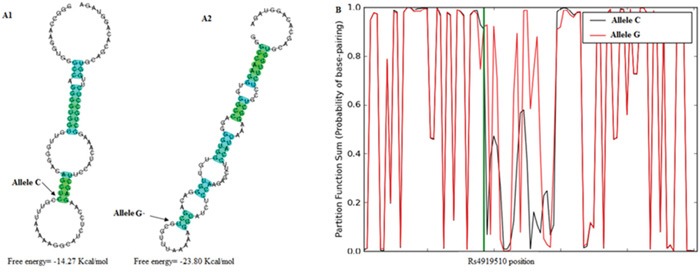
Bioinformatics prediction of the influence of rs4919510 on pre-miR-608 folding **A.** Secondary folding predicted by CentroidFold software; A1: The folding carrying allele C of the locus; A2: The folding carrying allele G of the locus; **B.** Secondary folding predicted by SNPFold software.

Using CentroidFold and SNPFold software, a significant difference was observed in the foldings of pre-miR-608 harbored rs4919510 allele C and G and the free energies of the two foldings were −14.27 and −23.80 Kcal/mol, respectively (Figure 3). However, no significant difference was found in the foldings and their free energies in pre-miR-124-1 and pre-miR-26a-1 carrying wild and mutant allele of rs531564 and rs7372209, respectively.

## DISCUSSION

In this study, we found a decreased susceptibility to 0-II stage CRC among patient harbored rs4919510 genotype GG and allele G, worse clinical RFS among patients with mutants of rs531564 and rs7372209 was observed in comparison with the genotype CC of the two loci, especially in patients undergoing adjuvant chemo-radiotherapy and II stage patients.

Recently several studies have reported the association between miR-608 rs4919510 and risk, survival in CRC [11–14], however the reported results of the studies with different population and sample size were inconsistent. No association was observed between the locus and risk of CRC in Caucasian and African Americans [10, 11]. However, CG and GG genotype of the locus were associated with poor survivals in CRC patients receiving 5-FU based chemotherapy in Caucasian population [11, 12]. Whereas, chemotherapy received CRC patients harbored mutants of the locus showed a better survival than those carrying the allele C and genotype CC in African Americans, Caucasian and Chinese populations, respectively [11, 13, 14]. In our study, the results showed that rs4919510 was only associated with a decreased risk in 0-II stage CRC in co-dominant, allele and recessive models, indicating that the locus was involved in early colorectal turmorigenesis and the genotype GG and allele G were susceptible factors for Chinese CRC population. Hsa-miR-608 was reported to be lowly expressed in colon cancer, chordoma, SW480, SW620 and A549 and SK-LU1 cell lines [15–17]. The polymorphism was located at the joint of stem with the canonical hairpin loop of pre-miR-608, and the locus might influence the recognition and processing by Drosha RNase, leading to decreased hsa-miR-608 [18]. Moreover, alternation of allele C to G of the locus changed pre-miR-608 secondary folding and the folding harbored allele G was more stable than it carrying allele C by *in silico* prediction. Additionally, *EGFR, BCL2I1, CD44* and *TUSC2P* were reported to be target genes of hsa-miR-608, the polymorphism influenced their binding efficacy, and resulted in inhibited expression of *EGFR, BCL2I1* and *CD44* oncogenes and increased translation of tumor suppressor gene *TUSC2* [14, 16, 19, 20]. Thus, rs4919510 of miR-608 might change the folding and stability of miR-608, and alter its target gene expression to involve in colorectal turmorigenesis.

In our study, we found pre-miR-124-1 rs531564 and pre-miR-26a-1 rs7372209 were associated with worse RFS in CRC undergoing chem-radiotherapy and stage II individuals, respectively, and the two unfavorable polymorphisms had cumulative effect on RFS in stage II patients. The results suggested that the polymorphisms were involved in CRC progression, and the patients harbored CC genotype of rs531564 could benefit from the adjuvant chemo-radiotherapy, stage II individuals carrying rs7372209 mutant genotype showed a worse RFS comparing to the CC genotype. Recently, rs531564 was reported to be inversely associated with risk of cervical cancer, esophageal squamous cell carcinoma in Chinese population [21, 22]. Rs7372209 was reported to associate with a significant increased risk of oral malignancy and a worse OS in advanced gastric cancer patients treated with chemotherapy [23, 24]. Although, we didn't observe association between the two loci and risk of CRC, our result was consistent with the finding of Boin et al [25]. Rs531564 and rs7372209 were allele C>G and C>T alternations locating in downstream 199bp of pre-miR-124-1 and upstream 187bp of pre-miR-26a-1, respectively. *In silico* prediction showed that the two loci didn't affect the secondary folding and stability of pre-miR-124-1 and pre-miR-26a-1. The two miRNAs were deemed as tumor suppressors in CRC, they were significantly down-regulated in HUES-17s and LoVo cell lines as well as colorectal adenoma and cancer in comparison with normal specimens [26–29]. Rs531564 was reported to alter the expression of hsa-miR-124-1 in HEK293T cells [30], thus mutant genotypes of the two loci might be associated with impaired expressions of hsa-miR-124-1 and hsa-miR-26a-1, respectively. The ectopic expression of miR-124-1 could induce apoptosis and autophagy in colon cancer cell [27]. Since miR-124-1 played a crucial role in DDX6/c-Myc/PTBI pathway and PTB1/PKM1/PKM2 feedback cascade, down-expression of its target genes *PTB1, ROCK1*, *KITENIN, STAT3* and *PRRX1* could inhibit CRC cell proliferation, motility, migration, invasion, tumor growth and enhance radio-sensitivity *in vivo* and *vitro* [26, 27, 31–34]. Moreover, miR-26a-1 could enhance miRNA biogenesis and regulation of glucose metabolism in CRC cell line by targeting *Lin28B, ZCCHC11, PDHX* to suppress CRC growth and metastasis [35, 36]. So, we believe that mutant genotypes of the two loci might contribute to low expression of hsa-miR-124-1 and hsa-miR-26a-1 and high expression of the target genes to implicate in CRC recurrence.

In summary, the findings of present study indicated that genotype CC and allele C of miR-608 rs4919510 could decrease predisposition to 0-II stage CRC, mutants of pre-miR-124-1 rs531564 and pre-miR-26a-1 rs7372209 increased recurrent risk in surgically resected CRC individuals receiving adjuvant chemo-radiotherapy and II stage patients, respectively. Further studies with a larger population with diverse ethnicity and functional experiment are warrant to confirm our findings in Chinese population.

## MATERIALS AND METHODS

### Included population

A total of 2437 peripheral blood samples including 1358 clinical newly diagnosed and histopathologically confirmed CRC patients and 1079 randomly selected healthy individuals without prior history of cancer were collected in this study. The included cases were all underwent surgical resection in Nanjing First Hospital and Xijing Hospital between April 2010 and August 2014. We collected the data of demographic characteristics, lifestyle, chronic disease and clinical pathological results from medical record of each participant. All of the enrolled individuals were Han Chinese population, which was consisted of more than 95% of the population. The design of present study was approved by Ethics Committee of Southeast University and we obtained each informed consent which was signed by each participant.

### SNP selection and genotyping

Common polymorphisms within miRNA involved in CRC turmorigenesis or metastasis were selected in accordance with the following included criteria: (1) miRNA was reported to associate with CRC carcinogenesis and prognosis; (2) SNP was located at mature sequence of hsa-miRNA, flanking sequence of pre-, pri-miRNA which showed to be of biological function by F-SNP database (http://compbio.cs.queensu. ca/F-SNP/) [37]; (3) frequency of minor allele was more than 5% in Chinese Han population; (4) there was no or few reports between the selected miRNA and CRC risk, drug response and prognosis; (5) the reported results concerning the selected SNP were contradictory. Consequently, a total of fifteen CRC related miRNA polymorphisms were retrieved, and only six polymorphsims (rs4919510 within miR-608, rs3746444 within miR-499a, rs2910164 within miR-146a, rs41291957 within pre-miR-143, rs531564 within pre-miR-124-1, rs7372209 within pre-miR-26a-1) were selected in accordance with the criteria. Human genomic DNA was extracted from each peripheral blood sample using Tiangen kit (Tiangen, Beijing, China) according to the manufacturer's protocol. Genotypes of all the selected SNPs were detected by PCR-based MassARRAY genotyping platform (Sequenom lnc, San Diego, USA). Meanwhile, 5% of random selected sample was tested for second time to validate the result.

### Assessment of therapeutic efficacy and follow-up

The assessment of response to 5-FU based first line chemotherapy was evaluated each month at the time of hospitalization in 276 CRC patients receiving neoadjuvant chemotherapy by two clinical professional physicians in accordance with the Response Evaluation Criteria in Solid Tumors 1.0. ORR, RFS and 3-years' OS were considered as endpoints in this study. The response to 5-FU based chemotherapy was defined as CR, PR, SD and PD. Follow-up was carried out every three months within three years in the cases who provided detail clinical data. Time from surgical operation to local and distant recurrence, death and the deadline of following up until up to June of 2015 were defined as RFS and OS, respectively.

### Bioinformatics prediction of SNP on miRNA folding

Due to SNP locating in flank region of pre-miRNA or mature miRNA, it may influence the biogenesis of mature miRNA, its secondary folding or stability. Thus, CentroidFold (http://www.ncrna.org/centroidfold/) and SNPfold (http://ribos-nitch.bio.unc.edu/snpfold/SNPfold.html) algorithms were selected to predict the putative influence of SNP on the selected pre-miRNA folding [38].

### Statistics

Pearson chi-square test was selected to compare the distributions of demographic, clinical characteristics and genotypes of each SNP between two groups and difference in clinical response to 5-FU based adjuvant chemotherapy according to genotypes of the SNPs in the cases. Kolmogorov-Smirnow test was used to analysis whether the continuous variables were normally distributed. The difference in continuous variables was compared using Student's t-test. Arlequin v3.0 software (http://cmpg.unibe.ch/software/arlequin3) was used to evaluate the departure of HWE in each SNP genotype distribution in two groups [39]. Binary logistic regression, Kaplan-Meier curve with log-rank test and back elimination multivariate Cox regression were selected to examine the association between the selected SNPs and risk, clinical outcome of the patients. OR and HR as well as 95%CI were used as common measurements to assess the strengths between them, respectively. The statistics were conducted using SPSS v17.0 (SPSS Inc., Chicago, IL), and *p*-value<0.05 was considered as statistical significance in all statistics. GraphPad Prism 5.0 software (GraphPad Software lnc., La Jolla, CA) was used for drawing in this study.

## SUPPLEMENTARY TABLES




