# Supplementary material for: MiR-608, pre-miR-124-1 and pre-miR26a-1 polymorphisms modify susceptibility and recurrence-free survival in surgically resected CRC individuals
Source: Oncotarget. 2016 Oct 4;7(46):75865–73. doi: 10.18632/oncotarget.12422 (PMC5342784; doi:10.18632/oncotarget.12422)
Supplement: Supplementary file 1 [file oncotarget-07-75865-s001.pdf]

MiR-608, pre-miR-124-1 and pre-miR26a-1 polymorphisms modify susceptibility and recurrence-free survival in surgically resected CRC individuals

SUPPLEMENTARY TABLES

Supplementary Table S1: Clinical demographic and histological characteristics in cases and controls

Variables		Cases (1358)	Controls (1079)	*P*-value*
		No. (%)	No. (%)	
Gender	Male	805(59.27%)	618(57.27%)	0.32
Age	Mean ± SD	60.80 ±13.03	60.70 ±15.61	0.87
Smoking	Yes	265(19.51%)	181(16.77%)	0.08
Drinking	Yes	185(13.62%)	154(14.27%)	0.65
Diabetes	Yes	239(17.60%)	177(16.40%)	0.44
Hypertension	Yes	206(15.16%)	157(14.55%)	0.67
Location	Proximal colon	295(21.72%)		
	Distal colon	285(20.99%)		
	Rectum	778(57.29%)		
TNM stage	0-I	191(14.07%)		
	II	592(43.59%)		
	III	575(42.34%)		
Invasive depth	Tis+T1	57(4.19%)		
	T2	149(10.97%)		
	T3	833(61.34%)		
	T4	319(23.49%)		
Node metastasis	N0	783(57.66%)		
	N1	395(29.09%)		
	N2	180(13.25%)		
Differentiation	Good	241(17.75%)		
	Median	946(69.66%)		
	Poor	171(12.59%)		
Surgical resection	Yes	1358(100.00%)		
5-FU based chemotherapy	Yes	1179(86.82%)		
Radiotherapy	Yes	1048(77.20%)		
Efficacy response	CR	2(0.73%)		
	PR	46(16.73%)		
	SD	161(58.55%)		
	PD	67(23.99%)		
Survival	Recurrence	524(43.02%)		
	Death	336(27.59%)		
	Alive	882(72.41%)		

Abbreviation: CR: complete response; PR: partial response; SD: stable disease; PD: progressive disease.
*Continuous and dichotomous variables were calculated by Student's t-test and Pearson χ^2 test, respectively.

Supplementary Table S2: Genotype distributions of the six miRNA polymorphisms in case and control groups

See Supplementary File 1

Supplementary Table S3: The six selected polymorphisms and clinical therapeutic efficacy in surgical resected CRC patients receiving 5-FU based chemotherapy

See Supplementary File 1

Supplementary Table S4: The six polymorphisms and clinical survival in 1218 surgically resected CRC patients

Locus	Genetic model	Comparison	Recurrence-free survival			3 year's overall survival		
			K-M	HR and 95%CI		K-M	HR and 95%CI	
			P-value	[1]	[2]	*P*-value	[1]	[2]
Rs531564	Co-dominant	CG vs.CC	0.10	1.18(0.97-1.44)	**1.25(1.02-1.52)**	0.62	1.06(0.83-1.36)	1.17(0.91-1.51)
		GG vs. CC	**0.03**	**1.66(1.03-2.66)**	1.54(0.96-2.48)	0.42	1.28(0.70-2.34)	1.26(0.68-2.31))
	Allele	G vs. C	**0.01**	**1.23(1.04-1.45)**	**1.26(1.07-1.48)**	0.40	1.09(0.89-1.35)	1.17(0.95-1.44)
	Dominant	CG/GG vs. CC	**0.03**	**1.22(1.01-1.48)**	**1.28(1.05-1.54)**	0.50	1.09(0.86-1.38)	1.19(0.93-1.51)
	Recessive	GG vs.CC/CG	**0.05**	**1.59(1.00-2.55)**	1.47(0.92-2.36)	0.45	1.26(0.69-2.30)	1.23(0.67-2.25)
	Over-dominant	CG vs.CC/GG	0.14	1.16(0.95-1.41)	**1.22(1.00-1.49)**	0.68	1.05(0.82-1.35)	1.16(0.90-1.49)
Rs7372209	Co-dominant	CT vs. CC	**0.05**	**1.19(1.00-1.42)**	1.16(0.97-1.39)	0.49	1.08(0.87-1.35)	1.06(0.84-1.32)
		TT vs. CC	0.27	0.81(0.55-1.19)	0.79(0.54-1.16)	0.40	0.81(0.50-1.32)	0.80(0.49-1.30)
	Allele	T vs. C	0.17	1.13(0.95-1.34)	1.10(0.93-1.31)	0.91	0.99(0.83-1.18)	0.98(0.82-1.16)
	Dominant	CT/TT vs. CC	0.65	1.03(0.90-1.19)	1.01(0.88-1.16)	0.72	1.04(0.84-1.29)	1.02(0.82-1.27)
	Recessive	TT vs. CC/CT	0.13	0.75(0.52-1.09)	0.73(0.50-1.07)	0.32	0.79(0.49-1.27)	0.79(0.49-1.27)
	Over-dominant	CT vs. CC/TT	**0.03**	**1.22(1.02-1.45)**	**1.20(1.00-1.42)**	0.37	1.10(0.89-1.38)	1.08(0.87-1.35)
Rs2910164	Co-dominant	CG vs. CC	0.60	1.05(0.87-1.28)	1.04(0.85-1.26)	0.56	1.07(0.85-1.36)	1.06(0.84-1.35)
		GG vs. CC	0.24	1.16(0.90-1.50)	1.17(0.91-1.52)	0.90	1.02(0.74-1.42)	1.08(0.77-1.50)
	Allele	G vs. C	0.26	1.07(0.95-1.21)	1.07(0.95-1.22)	0.78	1.02(0.88-1.19)	1.04(0.89-1.22)
	Dominant	CG/GG vs. CC	0.40	1.08(0.90-1.29)	1.07(0.89-1.29)	0.61	1.06(0.85-1.33)	1.08(0.86-1.35)
	Recessive	GG vs. CC/ CG	0.30	1.13(0.90-1.41)	1.15(0.91-1.44)	0.90	0.98(0.73-1.32)	1.02(0.76-1.38)
	Over-dominant	CG vs. CC/ GG	0.97	1.00(0.84-1.19)	0.99(0.83-1.18)	0.56	1.07(0.86-1.32)	1.06(0.85-1.31)
Rs4919510	Co-dominant	CG vs. CC	0.37	0.92(0.76-1.11)	0.93(0.76-1.13)	0.32	0.89(0.70-1.13)	0.89(0.70-1.14)
		GG vs. CC	0.77	1.04(0.81-1.34)	1.08(0.83-1.40)	0.87	1.03(0.75-1.41)	1.10(0.80-1.52)
	Allele	G vs. C	0.99	1.00(0.89-1.13)	1.02(0.90-1.16)	0.91	0.99(0.85-1.16)	1.02(0.87-1.19)
	Dominant	CG/GG vs. CC	0.54	0.94(0.79-1.14)	0.97(0.80-1.16)	0.46	0.92(0.73-1.15)	0.94(0.75-1.18)
	Recessive	GG vs. CG/ CC	0.42	1.10(0.87-1.38)	1.14(0.91-1.43)	0.47	1.11(0.84-1.47)	1.18(0.89-1.57)
	Over-dominant	CG vs. GG/ CC	0.24	0.90(0.76-1.07)	0.90(0.76-1.07)	0.23	0.88(0.71-1.09)	0.87(0.70-1.08)
Rs41291957	Co-dominant	GA vs. GG	0.09	0.85(0.71-1.03)	0.85(0.71-1.03)	0.13	0.84(0.67-1.06)	0.85(0.67-1.07)
		AA vs. GG	0.65	1.07(0.80-1.43)	1.13(0.83-1.49)	0.76	1.06(7.40-1.51)	1.07(0.75-1.53)
	Allele	A vs. G	0.57	0.96(0.84-1.10)	0.98(0.86-1.12)	0.58	0.96(0.81-1.13)	0.97(0.82-1.15)
	Dominant	GA/AA vs. GG	0.19	0.89(0.75-1.06)	0.90(0.76-1.07)	0.24	0.88(0.71-1.09)	0.89(0.72-1.11)
	Recessive	AA vs. GA/ GG	0.32	1.15(0.87-1.52)	1.22(0.92-1.62)	0.45	1.14(0.81-1.60)	1.18(0.84-1.67)
	Over-dominant	GA vs. AA/ GG	0.06	0.84(0.70-1.00)	0.84(0.70-1.00)	0.10	0.83(0.67-1.04)	0.83(0.66-1.04)
Rs3746444	Co-dominant	AG vs. AA	0.38	0.80(0.59-1.16)	0.79(0.55-1.62)	0.90	0.98(0.73-1.32)	1.02(0.76-1.38)
		GG vs. AA	0.41	1.21(0.79-1.56)	1.18(0.87-1.44)	0.58	1.07(0.88-1.32)	1.16(0.85-1.38)
	Allele	G vs. A	0.55	1.17(0.84-1.46)	1.12(0.84-1.36)	0.47	1.07(0.95-1.21)	1.07(0.95-1.22)
	Dominant	AG/GG vs. AA	0.92	1.12(0.74-1.52)	1.13(0.77-1.52)	0.41	1.01(0.90-1.31)	1.04(0.89-1.29)
	Recessive	GG vs.AG/AA	0.78	1.12(0.87-1.29)	1.03(0.90-1.22)	0.30	1.12(0.89-1.41)	1.14(0.90-1.45)
	Over-dominant	AG vs. GG/ AA	0.81	1.12(0.94-1.56)	1.18(0.87-1.45)	0.80	1.22(0.77-1.54)	1.21(0.77-1.65)

Abbreviation: K-M: Kaplan-Meier curve; HR and 95%CI: hazard ratio and 95% confidential interval; *p*-value: the result of log-rank test of Kaplan-Meier curve; [1]: crude HR and 95%CI; [2]: adjusted by gender, age, smoking, drinking, and hypertension as well as diabetes; the bold highlighted results showed statistical significance.

Supplementary Table S5: Rs531564 within pre-miR-124-1, rs7372209 within pre-miR-26a-1 and RFS in subgroups stratified by treatment after surgical resection

Locus	Genetic model	Comparison	Recurrence-free survival			
			Untreated by adjuvant therapy		Treated by chemo-radiotherapy	
			K-M	HR and 95%CI	K-M	HR and 95%CI*
Rs531564	Co-dominant	CG vs.CC	0.79	1.10(0.42-2.88)	**0.05**	**1.24(1.01-1.52)**
		GG vs. CC	-		0.02	1.58(0.97-2.58)
	Allele	G vs. C	0.76	1.18(0.54-2.61)	**0.01**	**1.26(1.07-1.49)**
	Dominant	CG/GG vs. CC	0.77	1.15(0.47-2.81)	**0.02**	**1.28(1.05-1.55)**
	Recessive	GG vs. CC/CG	-		0.04	1.54(0.94-2.48)
	Over-dominant	CG vs. CC/GG	0.79	1.09(0.42-2.85)	0.07	1.22(0.99-1.49)
Rs7372209	Co-dominant	CT vs. CC	0.07	1.63(0.89-2.96)	0.15	1.14(0.94-1.37)
		TT vs. CC	-		0.31	0.82(0.55-1.23)
	Allele	T vs. C	0.57	0.95(0.60-1.50)	0.81	1.01(0.88-1.17)
	Dominant	CT/TT vs. CC	0.19	1.23(0.690-2.22)	0.32	1.09(0.91-1.30)
	Recessive	TT vs. CC/CT	-		0.18	0.76(0.51-1.13)
	Over-dominant	CT vs. CC/TT	**0.05**	1.65(0.92-2.97)	0.09	1.16(0.97-1.40)

Abbreviation: K-M: Kaplan-Meier curve; HR and 95%CI (hazard ratio and 95% confidential interval)*: adjusted by gender, age, smoking, drinking, and hypertension as well as diabetes; the bold highlighted results showed statistical significance.

Supplementary Table S6: Association of rs531564 within pre-miR-124-1, rs7372209 within pre-miR-26a-1 with clinical pathological characteristics in cases

Characteristics		Rs531564								Rs7372209							
		Genotype and allele					*P*-value*			Genotype and allele					*P*-value**		
		CC	CG	GG	C	G	[1]	[2]	[3]	CC	TC	TT	C	T	[1]	[2]	[3]
TNM stage	0-I	130	57	4	317	65				109	69	13	287	95			
	II	424	147	15	995	177	0.21	0.81	0.37	309	228	46	846	320	0.39	0.51	0.32
	III	428	134	11	990	156	0.07	0.76	0.10	319	217	34	855	285	0.69	0.74	0.96
Differentiation	Good	180	52	9	412	70				127	101	13	355	127			
	median	673	249	21	1595	291	0.15	0.24	0.62	515	353	66	1383	485	0.33	0.48	0.86
	Poor	128	37	1	293	39	1.00	0.05	0.25	95	60	14	250	88	0.28	0.37	0.92
Invasion depth	Tsi-T2	142	60	4	344	68				114	78	14	306	106			
	T3-T4	840	278	36	1958	350	0.15	0.43	0.49	623	436	79	1682	594	0.89	0.92	0.88
Node metastasis	N0	554	204	19	1312	242				418	297	59	1133	415			
	N1-N2	428	134	11	990	156	0.21	0.45	0.16	319	217	34	855	285	0.71	0.22	0.29

P-value*: [1]: genotype CG vs. CC; [2]: genotype GG vs. CC; [3]: allele G vs. C; *P*-value**: [1]: genotype TC vs. CC; [2]: genotype TT vs. CC; [3]: allele T vs. C.
