# Supplementary material for: MiR-608, pre-miR-124-1 and pre-miR26a-1 polymorphisms modify susceptibility and recurrence-free survival in surgically resected CRC individuals
Source: Oncotarget. 2016 Oct 4;7(46):75865–73. doi: 10.18632/oncotarget.12422 (PMC5342784; doi:10.18632/oncotarget.12422)
Supplement: Supplementary file 2 [file oncotarget-07-75865-s002.doc]

**Supplementary Table 2** Genotype distributions of the six miRNA polymorphisms in case and control groups.

| **SNP** | **MicroRNA** | **Genetic model** | **Genotype** | **Cases** | **Controls** | **HWE** | ***P*-value** | **OR and 95%CI** | |
| --- | --- | --- | --- | --- | --- | --- | --- | --- | --- |
|  |  |  |  |  |  |  |  | **[1]** | **[2]** |
| Rs531564 | Pre-miR-124-1 | Co-dominant | CC | 982 | 779 | Cases: | 1.00 | 1.00 | 1.00 |
|  |  |  | CG | 338 | 276 | *P*=0.885; | 0.76 | 0.97(0.81-1.17) | 0.96(0.80-1.16) |
|  |  |  | GG | 30 | 24 | Controls: | 0.98 | 0.99(0.58-1.71) | 0.97(0.56-1.68) |
|  |  | Allele | C | 2302 | 1834 | *P*=0.939 | 1.00 | 1.00 | 1.00 |
|  |  |  | G | 398 | 324 |  | 0.79 | 0.98(0.84-1.15) | 0.97(0.83-1.14) |
|  |  | Dominant | CC | 982 | 779 |  | 1.00 | 1.00 | 1.00 |
|  |  |  | CG/GG | 368 | 300 |  | 0.77 | 0.97(0.81-1.16) | 0.96(0.80-1.15) |
|  |  | Recessive | CC/CG | 1320 | 1055 |  | 1.00 | 1.00 | 1.00 |
|  |  |  | GG | 30 | 24 |  | 1.00 | 1.00(0.58-1.72) | 0.99(0.58-1.71) |
|  |  | Over-dominant | CC/GG | 1012 | 803 |  | 1.00 | 1.00 | 1.00 |
|  |  |  | CG | 338 | 276 |  | 0.76 | 0.97(0.81-1.17) | 0.96(0.80-1.16) |
| Rs7372209 | Pre-miR-26a-1 | Co-dominant | CC | 737 | 582 | Cases: | 1.00 | 1.00 | 1.00 |
|  |  |  | CT | 514 | 432 | *P=*0.793; | 0.47 | 0.94(0.79-1.11) | 0.94(0.79-1.11) |
|  |  |  | TT | 93 | 65 | Controls: | 0.48 | 1.13(0.81-1.58) | 1.14(0.81-1.60) |
|  |  | Allele | C | 1988 | 1596 | *P=*0.196 | 1.00 | 1.00 | 1.00 |
|  |  |  | T | 700 | 562 |  | 1.00 | 1.00(0.88-1.14) | 1.00(0.88-1.14) |
|  |  | Dominant | CC | 737 | 582 |  | 1.00 | 1.00 | 1.00 |
|  |  |  | CT/TT | 607 | 497 |  | 0.66 | 0.96(0.82-1.13) | 0.96(0.82-1.13) |
|  |  | Recessive | CC/CT | 1251 | 1014 |  | 1.00 | 1.00 | 1.00 |
|  |  |  | TT | 93 | 65 |  | 0.38 | 1.16(0.84-1.61) | 1.16(0.84-1.61) |
|  |  | Over-dominant | CC/TT | 830 | 647 |  | 1.00 | 1.00 | 1.00 |
|  |  |  | CT | 514 | 432 |  | 0.37 | 0.93(0.79-1.09) | 0.92(0.78-1.09) |
| Rs2910164 | MiR-146a | Co-dominant | CC | 473 | 383 | Cases: | 1.00 | 1.00 | 1.00 |
|  |  |  | CG | 655 | 529 | *P*=0.882； | 0.98 | 1.00(0.84-1.20) | 1.00(0.84-1.19) |
|  |  |  | GG | 223 | 163 | Controls: | 0.41 | 1.11(0.87-1.41) | 1.10(0.87-1.41) |
|  |  | Allele | C | 1601 | 1295 | *P*=0.732 | 1.00 | 1.00 | 1.00 |
|  |  |  | G | 1101 | 855 |  | 0.49 | 1.04(0.93-1.17) | 1.04(0.93-1.17) |
|  |  | Dominant | CC | 473 | 383 |  | 1.00 | 1.00 | 1.00 |
|  |  |  | CG/GG | 878 | 692 |  | 0.75 | 1.03(0.87-1.22) | 1.03(0.87-1.21) |
|  |  | Recessive | CC/CG | 1128 | 912 |  | 1.00 | 1.00 | 1.00 |
|  |  |  | GG | 223 | 163 |  | 0.37 | 1.11(0.89-1.38) | 1.10(0.89-1.38) |
|  |  | Over-dominant | CC/GG | 696 | 546 |  | 1.00 | 1.00 | 1.00 |
|  |  |  | CG | 655 | 529 |  | 0.72 | 0..97(0.83-1.14) | 0.97(0.83-1.14) |
| Rs4919510 | MiR-608 | Co-dominant | CC | 423 | 313 | Cases: | 1.00 | 1.00 | 1.00 |
|  |  |  | CG | 690 | 512 | *P*=0.084; | 0.98 | 1.00(0.83-1.20) | 1.00(0.84-1.22) |
|  |  |  | GG | 232 | 250 | Control: | **<0.01** | **0.69(0.55-0.87)** | **0.70(0.55-0.88)** |
|  |  | Allele | C | 1536 | 1138 | *P*=0.148 | 1.00 | 1.00 | 1.00 |
|  |  |  | G | 1154 | 1012 |  | **<0.01** | **0.85(0.75-0.95)** | **0.85(0.75-0.95)** |
|  |  | Dominant | CC | 423 | 313 |  | 1.00 | 1.00 | 1.00 |
|  |  |  | CG/GG | 922 | 762 |  | 0.22 | 0.90(0.75-1.07) | 0.90(0.75-1.07) |
|  |  | Recessive | CC/CG | 1113 | 825 |  | 1.00 | 1.00 | 1.00 |
|  |  |  | GG | 232 | 250 |  | **<0.01** | **0.69(0.56-0.84)** | **0.68(0.56-0.86)** |
|  |  | Over-dominant | CC/GG | 655 | 563 |  | 1.00 | 1.00 | 1.00 |
|  |  |  | CG | 690 | 512 |  | 0.07 | 1.16(0.99-1.36) | 1.17(0.99-1.37) |
| Rs41291957 | Pre-miR-143 | Co-dominant | GG | 638 | 505 | Cases: | 1.00 | 1.00 | 1.00 |
|  |  |  | AG | 585 | 450 | *P*=0.700; | 0.74 | 1.03(0.87-1.22) | 1.03(0.87-1.22) |
|  |  |  | AA | 127 | 120 | Controls: | 0.21 | 0.84(0.64-1.10) | 0.84(0.64-1.11) |
|  |  | Allele | G | 1861 | 1460 | *P*=0.194 | 1.00 | 1.00 | 1.00 |
|  |  |  | A | 839 | 690 |  | 0.45 | 0.95(0.85-1.08) | 0.960(0.850-1.08) |
|  |  | Dominant | GG | 638 | 505 |  | 1.00 | 1.00 | 1.00 |
|  |  |  | AG/AA | 712 | 570 |  | 0.89 | 0.99(0.84-1.16) | 0.99(0.85-1.17) |
|  |  | Recessive | GG/GA | 1223 | 955 |  | 1.00 | 1.00 | 1.00 |
|  |  |  | AA | 127 | 120 |  | 0.16 | 0.83(0.64-1.08) | 0.83(0.64-1.09) |
|  |  | Over-dominant | GG/AA | 765 | 625 |  | 1.00 | 1.00 | 1.00 |
|  |  |  | AG | 585 | 450 |  | 0.47 | 1.06(0.90-1.25) | 1.06(0.90-1.25) |
| Rs3746444 | MiR-499a | Co-dominant | AA | 872 | 713 | Cases: | 1.00 | 1.00 | 1.00 |
|  |  |  | AG | 336 | 245 | *P*<0.05; | 0.24 | 1.12(0.93-1.36) | 1.13(0.91-1.36) |
|  |  |  | GG | 142 | 117 | Controls: | 0.96 | 0.99(0.76-1.29) | 0.99(0.76-1.30) |
|  |  | Allele | A | 2080 | 1671 | *P*<0.05 | 1.00 | 1.00 | 1.00 |
|  |  |  | G | 620 | 479 |  | 0.57 | 1.04(0.91-1.19) | 1.02(0.90-1.23) |
|  |  | Dominant | AA | 872 | 713 |  | 1.00 | 1.00 | 1.00 |
|  |  |  | AG/GG | 478 | 362 |  | 0.37 | 1.08(0.91-1.28) | 1.03(0.91-1.31) |
|  |  | Recessive | AA/AG | 1208 | 958 |  | 1.00 | 1.00 | 1.00 |
|  |  |  | GG | 142 | 117 |  | 0.77 | 0.96(0.74-1.25) | 0.97(0.75-1.25) |
|  |  | Over-dominant | AA/GG | 1014 | 830 |  | 1.00 | 1.00 | 1.00 |
|  |  |  | AG | 336 | 245 |  | 0.23 | 0.89(0.74-1.08) | 0.90(0.74-1.08) |

**Abbreviation:** HWE: Hardy-Weinberg equilibrium; OR: odds ratio; 95%CI: 95% confidence interval; [1]: crude OR and 95%CI; [2]: OR and 95%CI were adjusted by gender, age, status of smoking and drinking, diabetes and hypertension; the bold highlighted results showed statistical significance.

**Supplementary Table 3** The six selected polymorphisms and clinical therapeutic efficacy in surgical resected CRC patients receiving 5-FU based chemotherapy.

| **Locus** | **Genetic model** |  | **Objective response** | | | **OR and 95%CI** | |
| --- | --- | --- | --- | --- | --- | --- | --- |
|  |  |  | **Yes** | **No** | **P-value** | **[1]** | **[2]** |
| Rs531564 | Co-dominant | CC | 37 | 153 | 1.00 | 1.00 | 1.00 |
|  |  | CG | 7 | 69 | **0.04** | **0.42(0.18-0.99)** | 0.54(0.22-1.31) |
|  |  | GG | 2 | 4 | - | - | - |
|  | Allele | C | 81 | 375 | 1.00 | 1.00 | 1.00 |
|  |  | G | 11 | 77 | 0.23 | 0.66(0.34-1.30) | 0.84(0.42-1.70) |
|  | Dominant | CC | 37 | 153 | 1.00 | 1.00 | 1.00 |
|  |  | CG/GG | 9 | 73 | 0.09 | 0.51(0.23-1.11) | 0.67(0.30-1.51) |
|  | Recessive | CC/CG | 44 | 222 | 1.00 | 1.00 | 1.00 |
|  |  | GG | 2 | 4 | - | - | - |
|  | Over-dominant | CC/GG | 39 | 157 | 1.00 | 1.00 | 1.00 |
|  |  | CG | 7 | 69 | **0.04** | **0.41(0.17-0.96)** | 0.52(0.22-1.27) |
| Rs7372209 | Co-dominant | CC | 25 | 123 | 1.00 | 1.00 | 1.00 |
|  |  | CT | 19 | 93 | 0.99 | 1.00(0.52-1.93) | 0.95(0.48-1.85) |
|  |  | TT | 2 | 10 | 1.00 | 0.98(0.20-4.77) | 1.21(0.23-6.27) |
|  | Allele | C | 69 | 339 | 1.00 | 1.00 | 1.00 |
|  |  | T | 23 | 113 | 1.00 | 1.00(0.60-1.68) | 0.97(0.57-1.65) |
|  | Dominant | CC | 25 | 123 | 1.00 | 1.00 | 1.00 |
|  |  | CT/TT | 21 | 103 | 0.99 | 1.00(0.53-1.90) | 0.95(0.49-1.83) |
|  | Recessive | CC/CT | 44 | 216 | 1.00 | 1.00 | 1.00 |
|  |  | TT | 2 | 10 | 0.98 | 0.98(0.21-4.64) | 1.07(0.22-5.28) |
|  | Over-dominant | CC/TT | 27 | 133 | 1.00 | 1.00 | 1.00 |
|  |  | CT | 19 | 93 | 0.99 | 1.00(0.53-1.92) | 0.94(0.48-1.82) |
| Rs2910164 | Co-dominant | CC | 12 | 74 | 1.00 | 1.00 | 1.00 |
|  |  | CG | 21 | 117 | 0.62 | 1.21(0.57-2.58) | 1.01(0.45-2.28) |
|  |  | GG | 11 | 35 | 0.15 | 1.94(0.78-4.82) | 1.99(0.75-5.27) |
|  | Allele | C | 45 | 265 | 1.00 | 1.00 | 1.00 |
|  |  | G | 43 | 187 | 0.18 | 1.36(0.87-2.13) | 1.33(0.84-2.11) |
|  | Dominant | CC | 12 | 74 | 1.00 | 1.00 | 1.00 |
|  |  | CG/GG | 32 | 152 | 0.38 | 1.38(0.68-2.82) | 1.22(0.58-2.55) |
|  | Recessive | CC/CG | 33 | 191 | 1.00 | 1.00 | 1.00 |
|  |  | GG | 11 | 35 | 0.17 | 1.72(0.80-3.69) | 1.98(0.89-4.38) |
|  | Over-dominant | CC/GG | 23 | 109 | 1.00 | 1.00 | 1.00 |
|  |  | CG | 21 | 117 | 0.83 | 0.93(0.49-1.76) | 0.76(0.39-1.49) |
| Rs4919510 | Co-dominant | CC | 17 | 61 | 1.00 | 1.00 | 1.00 |
|  |  | CG | 20 | 122 | 0.42 | 0.68(0.27-1.73) | 0.93(0.34-2.58) |
|  |  | GG | 8 | 42 | 0.14 | 0.59(0.29-1.20) | 0.56(0.26-1.19) |
|  | Allele | C | 54 | 244 | 1.00 | 1.00 | 1.00 |
|  |  | G | 36 | 206 | 0.07 | 0.66(0.42-1.03) | 0.62(0.39-1.11) |
|  | Dominant | CC | 17 | 61 | 1.00 | 1.00 | 1.00 |
|  |  | CG/GG | 28 | 164 | 0.15 | 0.61(0.31-1.20) | 0.61(0.31-1.24) |
|  | Recessive | CC/CG | 37 | 183 | 1.00 | 1.00 | 1.00 |
|  |  | GG | 8 | 42 | 0.23 | 0.68(0.36-1.29) | 0.61(0.31-1.18) |
|  | Over-dominant | CC/GG | 25 | 103 | 1.00 | 1.00 | 1.00 |
|  |  | CG | 20 | 122 | 0.90 | 0.94(0.41-2.17) | 1.16(0.48-2.79) |
| Rs41291957 | Co-dominant | GG | 32 | 124 | 1.00 | 1.00 | 1.00 |
|  |  | GA | 14 | 82 | 0.24 | 0.66(0.33-1.32) | 0.51(0.25-1.06) |
|  |  | AA | 2 | 20 | 0.20 | 0.39(0.09-1.75) | 0.46(0.09-2.21) |
|  | Allele | G | 78 | 330 | 1.00 | 1.00 | 1.00 |
|  |  | A | 18 | 122 | 0.09 | 0.62(0.36-1.08) | 0.57(0.33-1.01) |
|  | Dominant | GG | 32 | 124 | 1.00 | 1.00 | 1.00 |
|  |  | GA/AA | 16 | 102 | 0.13 | 0.61(0.32-1.17) | 0.49(0.24-0.97) |
|  | Recessive | GG/GA | 46 | 206 | 1.00 | 1.00 | 1.00 |
|  |  | AA | 2 | 20 | 0.28 | 0.45(0.10-1.98) | 0.56(0.12-2.55) |
|  | Over-dominant | GG/AA | 34 | 144 | 1.00 | 1.00 | 1.00 |
|  |  | GA | 14 | 82 | 0.35 | 0.72(0.37-1.43) | 0.53(0.25-1.09) |
| Rs3746444 | Co-dominant | AA | 32 | 153 | 1.00 | 1.00 | 1.00 |
|  |  | AG | 11 | 49 | 0.86 | 0.93(0.44-1.99) | 0.94(0.43-2.01) |
|  |  | GG | 5 | 25 | 0.93 | 1.05(0.37-2.94) | 1.04(0.37-2.94) |
|  | Allele | A | 75 | 355 | 1.00 | 1.00 | 1.00 |
|  |  | G | 21 | 99 | 0.99 | 1.00(0.59-1.70) | 1.00(0.59-1.70) |
|  | Dominant | AA | 32 | 153 | 1.00 | 1.00 | 1.00 |
|  |  | AG/GG | 16 | 74 | 0.92 | 0.97(0.50-1.87) | 0.99(0.54-1.90) |
|  | Recessive | AA/AG | 43 | 202 | 1.00 | 1.00 | 1.00 |
|  |  | GG | 5 | 25 | 0.90 | 1.06(0.39-2.94) | 1.01(0.39-2.96) |
|  | Over-dominant | AG | 11 | 49 | 1.00 | 1.00 | 1.00 |
|  |  | AA/GG | 37 | 178 | 0.84 | 0.93(0.44-1.95) | 0.93(0.46-1.95) |

**Abbreviation:** OR: odds ratio; 95%CI: 95% confidential interval; [1]: crude OR and 95%CI; [2]: adjusted by gender, age, smoking, drinking, hypertension and diabetes; the bold highlighted results showed statistical significance.
